# Deep sequencing of human papillomavirus positive loco-regionally advanced oropharyngeal squamous cell carcinomas reveals novel mutational signature

**DOI:** 10.1186/s12885-018-4567-3

**Published:** 2018-06-07

**Authors:** Christian Grønhøj, David H. Jensen, Tina Agander, Katalin Kiss, Estrid Høgdall, Lena Specht, Frederik Otzen Bagger, Finn Cilius Nielsen, Christian von Buchwald

**Affiliations:** 1 Department of Otorhinolaryngology – Head and Neck Surgery and Audiology, Rigshospitalet, University of Copenhagen, Blegdamsvej 9, 2100 Copenhagen, Denmark; 2 Department of Pathology, Rigshospitalet, University of Copenhagen, Blegdamsvej 9, 2100 Copenhagen, Denmark; 3 Department of Pathology, Herlev Hospital, University of Copenhagen, Copenhagen, Denmark; 4 Department of Oncology, Rigshospitalet, University of Copenhagen, Copenhagen, Denmark; 5 Centre for Genomic Medicine, Rigshospitalet, University of Copenhagen, Copenhagen, Denmark

**Keywords:** Gene sequencing, Deep sequencing, HPV, Human papilloma virus, Oropharyngeal cancer

## Abstract

**Background:**

The genetic profile for human papilloma virus positive (HPV+) oropharyngeal squamous cell carcinomas (OPSCC) remains largely unknown. The purpose of this study was to sequence tissue material from a large cohort of locoregionally-advanced HPV+ OPSCCs.

**Methods:**

We performed targeted deep sequencing of 395 cancer-associated genes in 114 matched tumor/normal loco-regionally advanced HPV+ OPSCCs. Mutations and copy number aberrations were determined.

**Results:**

We identified a total of 3459 mutations with an average of 10 mutations per megabase and a median of 28 variants per sample. The most frequently mutated genes were *KALRN* (28%), *SPTBN1* (32%), *KMT2A* (31%), *ZNRF3* (9%), *BNC2* (12%), *NOTCH2* (25%), *FGFR2* (12%), *SMAD2* (6%), and *AR* (13%). Our findings were dominated by COSMIC signature 5 and 12, represented in other head and neck cancers and in hepatocellular carcinomas, respectively.

**Conclusions:**

We have identified multiple genetic aberrations in HPV+ OPSCCs, and the COSMIC signature 12 as most prevalent. The mutations harbour both therapeutic and prognostic potential.

**Electronic supplementary material:**

The online version of this article (10.1186/s12885-018-4567-3) contains supplementary material, which is available to authorized users.

## Background

Head and neck squamous cell carcinoma (HNSCC) is among the most prevalent cancers worldwide partly due to the growing number of oropharyngeal squamous cell carcinomas (OPSCCs) associated with human papillomavirus infection (HPV+) [1, 2]. Compared with the HPV-negative OPSCCs, this subset of cancer is associated with favourable outcome likely explained by a difference in immunological [3], clinical [4, 5], and histopathological features [6]. Consequently, the interest in the mutational profile of HPV-associated HNSCCs is growing.

High-throughput DNA sequencing has led to identification of alterations in genes and pathways involved in the tumorigenic processes. With the exception of two smaller studies addressing structural DNA changes and mutations in HPV+ OPSCCs [7–9], large-scale studies have primarily mapped the diverse genetic landscape of HPV negative HNSCCs [10, 11]. Unlike a number of adenocarcinomas, no targetable genetic aberrations for OPSCCs are approved for treatment or as prognostic biomarkers. OPSCC patients are generally treated according to stage; typically for advanced disease with radiation, chemotherapy, or both, and for low stage disease surgery. The anti-EGFR-antibody (e.g. cetuximab) is the only approved targeted therapy but is has shown low to moderate effect in single-drug trials, and no convincing results as predictive biomarker [12–14].

Our knowledge about the mutational profile for HPV+ OPSCCs is incomplete, but carries the prospect of identification of targets for drug intervention as well as prognostic biomarkers for patient stratification in trial design. The purpose of this study is to present a comprehensive assessment of genetic aberrations in loco-regionally advanced HPV+ OPSCCs.

## Methods

We included 114 matched tumor- and normal tissues from patients diagnosed with a HPV+ OPSCC in eastern Denmark [1, 15, 16]. The patients were identified through the Danish Head and Neck Cancer group (DAHANCA) database and validated through the national Danish Pathology Data Registry (DPDR). An expert head and neck pathologist validated the diagnosis of OPSCC from a hematoxylin and eosin (H&E-) stained section of each tumour. The p16-staining was considered positive if a strong and diffuse nuclear and cytoplasmic reaction was present in more than 75% of the tumour cells [17]. Formalin-fixed paraffin embedded (FFPE) tumour specimens was handled according to standard operating procedures for the p16 immunohistochemistry. The protocol for p16 immunohistochemistry and HPV DNA PCR is previously described in detail [1, 16]. Smoking was quantified in number of pack-years (one pack year equals 20 cigarettes per day for one year), and data was collected from medical files. From an H&E-stained section, tumor and normal tissue was contoured by a head and neck pathologist. Tumor and normal tissue was subsequently punched biopsied from the FFPE-block to avoid contamination.

### Sequencing data generation and analysis

A custom panel of 395 cancer-associated genes (Additional file 1: Table S1) were targeted and sequenced in tumour-normal pairs. Genes were identified via cBioportal.org and selected based on results from The Cancer Genome Atlas [18], Agrawal et al. [19], and Rubio-Perez et al. [20]. Median coverage of the targeted bases was 150X in the tumours and 200X in the matched normal tissue.

### DNA extraction

Deamination of cytosine bases to uracil is a known source of DNA damage occurring in FFPE blocks that may lead to cytosine-thymine conversion (artefacts) in PCR and/or sequencing reactions [21], that can be ameliorated with the use of an Uracil-N-Glycosylase [22]. Therefore, we used the commercial GeneRead DNA FFPE Kit (Qiagen, Hilden, Germany), which is based on specific removal of deaminated cytosine residues from FFPE DNA using the Uracil-N-Glycosylase enzyme. The kit was used with the manufacturer’s instructions, except for the use of double amount of proteinase K and deparaffination solution, and samples were left overnight for proteinase K digestion at 56 °C.

### Library preparation and sequencing

Genome libraries were prepared according to the Ovation Custom Target Enrichment System protocol (NuGen). Landing-probes were designed by NuGEN to target the chosen genes. Target enrichment and library preparation was done according to the manufacturer’s protocol (NuGEN Technologies, San Carlos, CA). Briefly, genomic DNA was fragmented, end-repaired and ligated with forward barcoded adaptors, followed by a bead purification step. The barcoded adaptor contained both an 8-nt sample-specific barcode and a 6-nt UMI. The latter was used to identify and remove duplicate reads. The reverse adaptor was annealed to the target regions and extended. The library amplification step comprised 25 PCR cycles followed by bead purification and sequencing. Sequencing was carried out on a NextSeq 500 (Illumina Inc., San Diego, CA) with single-end sequencing (150-bp) using the high-output kit v2.

### Bioinformatics analysis

Raw sequencing data (.bcl files) were demultiplexed into individual FastQ read files with Illumina’s bcl2fastq v2.16.0.10 (Illumina Inc., San Diego, CA) based on their unique index, with the R1 read containing the forward read and R2 containing the UMI. Each sequencing library was checked for quality with fastQC (version 0.11.5). A combination of cutadapt (version 1.11), BBDuk (version 35.82) and ERNE-filtering (version 2.1.1) was employed to remove adapter and linker sequences, remove trailing probe sequence on R1, and clip low-quality bases ends. Read alignment was carried out with the BWA algorithm (BWA version 0.7.10) with UCSC hg19 (GRCh37) as the reference. Deduplication was performed with an *in-house* script developed at IGA Technology (http://igatechnology.com), which makes use of the UMI to accurately remove PCR duplicates. The deduplicated reads were subsequently prepared for variant calling after realignment to correct for misalignments around indels and recalibrating base qualities based on known polymorphisms present in the human dbSNP (dbSNP137) with the GATK tool (v3.6–0-g89b7209). Variants were called with Mutect2 based on tumour-normal pairs with standard settings. Only mutations that passed Mutect2 filters were used in the downstream analysis. We performed a filtering step before annotation, where SNPs present in the European 1000 Genomes cohort [23] with an allele frequency above 1% were removed, as they are more likely to represent common population polymorphisms than somatic mutations. We also filtered SNPs that did not full-fill the following criteria: Number of mutant allele reads in tumour sample ≥ 5, total number of allele reads ≥20, quality score (QSS) > 20 and variant allele fraction > 0.1, in order better to exclude C > T variants associated with FFPE artefacts [24]. To account for false positive variants from Mutect2 samples with a SQSS > 20 was filtered, where SQSS is QSS/allelic depth in the tumour [25]. For annotation, we used Oncotator v. 1.8.1.0 [26]. For visualization of mutations we used maftools [27]. Somatic genes that were significantly mutated were identified using OncodriveFML 1.1 using a threshold of q < 0.1 with CADD v.1.3, a signature by cancer type and coding regions [28]. Mutational signatures were predicted in each sample using the COSMIC framework [29]. Substitution mutations across the whole genome were analysed in context of the flanking nucleotides (96 possible trinucleotide combinations). Identified signatures were compared with 30 other validated signatures, and the frequency of each signature per megabase was determined. The numbers of enriched mutational signatures were established by a best-fit rank based on decreasing cophenetic correlation coefficients. Apolipoprotein B mRNA editing enzyme, catalytic polypeptide-like (APOBEC) enrichment scores were estimated as described by Burns et al. [30]. Briefly, enrichment of C > T mutations occurring within a tri-nucleotide DNA sequence (tCw) motif over all of the C > T mutations in a given sample was compared to background cytosine ratio and tCw’s occurring within 20 bp of mutated bases. Samples were classified as APOBEC-enriched based on an APOBEC enrichment score > 2 and/or false discovery rate (FDR) < 0.05. DNA copy number variations (CNV) were predicted by analysing the panel-sequencing data using CNVPanelizer, which compares tumour samples with a pool of normal tissue samples. The algorithm combines bootstrapping the reference set with the subsampling of amplicons associated with each of the target genes. This serves as a non-parametric estimation of the distribution of the gene-wise mean ratio between healthy reference samples and each tumour sample. All normal samples were used as a reference for calculating the sample-wise CNVs. Only genes that passed the noise and significance filter are reported as amplifications or deletions. To compare the difference in mutations between patients with high and low exposure to tobacco, we performed a fisher test on all genes between the two conditions to detect differentially mutated genes.

## Results

**Fig. 1:**
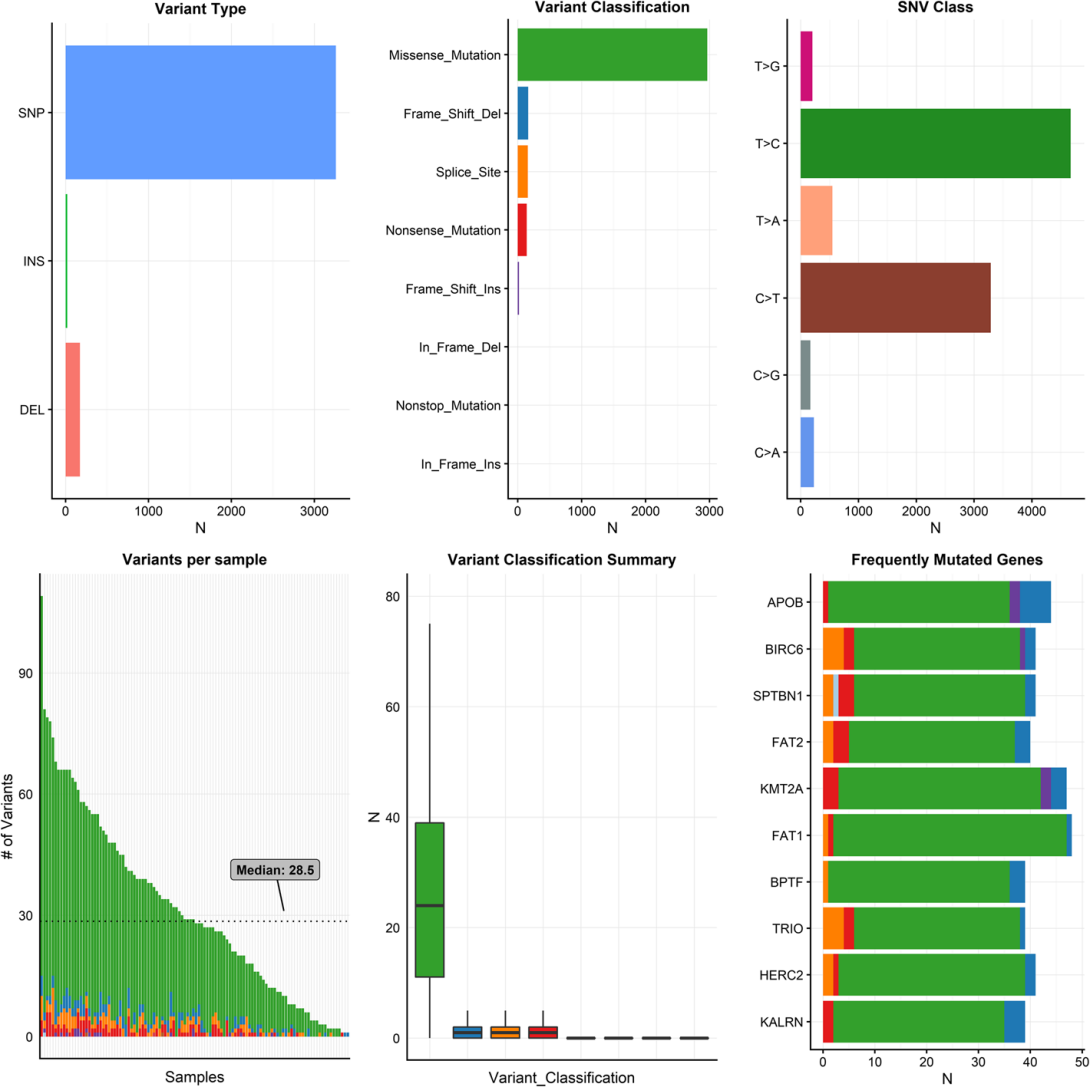
Top left: Number of mutations by type. Top middle: number of mutations by mutation type. Top right: Number of SNVs by type of variation. Bottom left: Number of variants per sample. Bottom middle: Type of variation. Bottom right: Top 10 mutated genes and type of mutations seen. Abbreviations: SNP: Single nucleotide polymorphism, INS, insertion, DEL, deletion. Green colour (missense mutation). Blue colour (Frame shift deletion). Orange (Splice site mutation). Red colour (nonsense mutation). Purple (Frame shift insertion)

We sequenced 114 locoregionally-advanced HPV-positive oropharyngeal squamous cell carcinomas. The targeted sequencing from 395 genes identified a total of 3459 mutations with an average of 10 mutations per megabase (excluding silent variants). The majority of mutations were missense mutations (86%), and less common mutations were frame-shift-deletions (5%), splice-site mutations (5%) and non-sense mutations (4%) (Fig. 1). The majority of identified mutations were SNPs, but deletions and insertions (indels) were also identified (Fig. 1). Of the 114 tumours 96% (*n* = 110) were HPV+/p16+ and the remaining HPV+/p16-.

### Mutation signatures

**Fig. 2:**
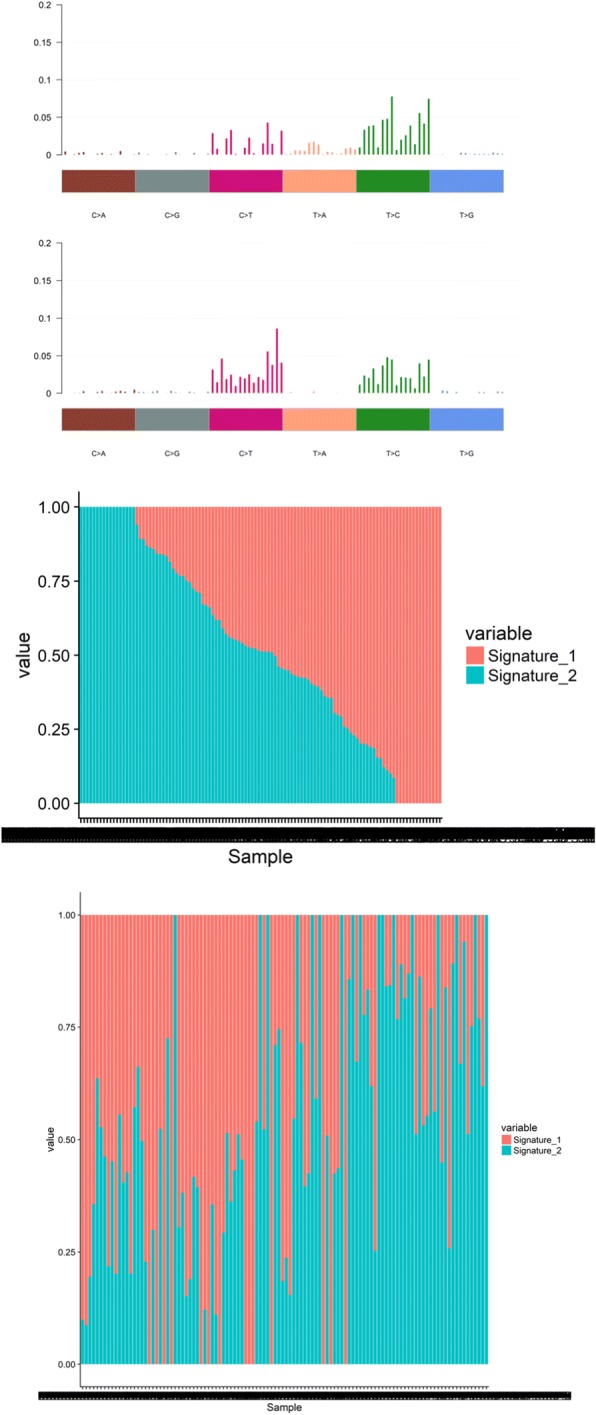
Top panels: Percentage of mutations belonging to the specified type of variant and percentage of transitions (Ti) and transversions (Tv). Lower panels: Contribution the signatures for each patient

**Fig. 3:**
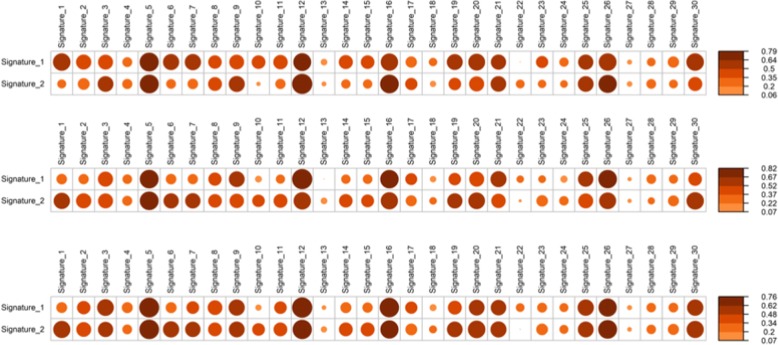
Panel (Top Panel) Correlation plots for signatures for all patients. Panel (Middle Panel) Correlation plots for signatures for patients with high tobacco smoking consumption. Panel (Lower Panel) Correlation plots for signatures for patients with low tobacco smoking consumption

The predominant type of SNV was found to be C > T and T > C (Figs. 1 and 2). The DNA substitution mutations were most commonly transitions (Fig. 2). The mutational pattern of each sample was compared against all 30 validated mutational signatures from COSMIC [29]. This was assessed by the COSMIC algorithm that takes the sequence context of each mutation into account (Fig. 3). The tumours were dominated by COSMIC signature 5 or 12 (Figs. 2 and 3). Signature 5 has previously been observed to be enriched in HNSCC. The signature is depicted in most cancers and exhibits transcriptional strand bias for T > C substitutions at an ApTpN context [31]. Signature 12 is known to be present in a subset of liver cancers, and exhibits a strong transcriptional strand-bias for T > C substitutions [31]. It was also investigated whether an APOBEC signature was significantly enriched in our samples. The APOBEC signature was enriched in a quarter of the samples (23%) [30] (Additional file 2: Figure S1).

### Somatic driver mutations

**Fig. 4:**
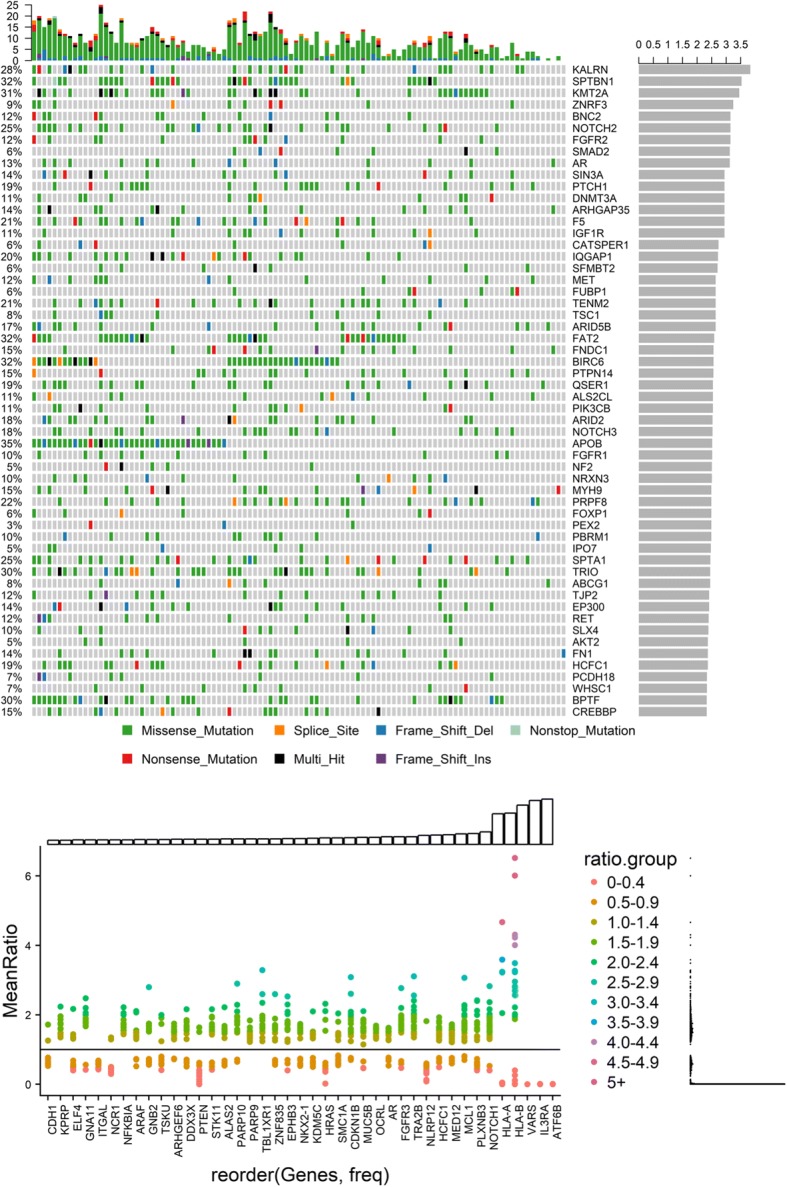
Mutations in significantly mutated genes from OncodriveFML sorted by q-value. Top panel: Total coding mutations per sample. Right: -10log(q-value). Lower panel: Top Panel Type of mutation with frequency in cohort given at the left. Lower Panel The most frequent deletions and copy number alterations in the cohort from CNVpanelizer. At the top of this panel is a histogram depicting the frequency of the alteration in the entire cohort

The OncodriveFML (q < 0.05) tool was employed to identify genes enriched in mutations. This algorithm identified 167 significantly mutated genes (Fig. 4). Of these *APOB* (35%), *BIRC6* (32%), *SPTBN1* (32%), *FAT2* (32%), *KMT2A* (31%), *FAT1* (30%), *BPTF* (30%), *TRIO* (30%), *HERC2* (28%), and *KALRN* (28%) were among the most frequently mutated genes. The most prevalent genomic alterations were located in *KALRN* (28%), *SPTBN1* (32%), KMT2A (31%), *ZNRF3* (9%), *BNC2* (12%), *NOTCH2* (25%), *FGFR2* (12%), *SMAD2* (6%), and *AR* (13%).

### Copy-number alterations

We determined CNAs from our sequencing panel by employing CNVPanelizer, an algorithm specifically designed to estimate copy number frequencies using targeted massive parallel sequencing data. The most frequent gene copy gain was detected for *ATF68* (*n* = 83; 72% of samples) and *IL3RA* (*n* = 81; 71% of samples). We found a high level of inter-patient CNA heterogeneity, but also a high intra-patient CN heterogeneity.

### Mutant-allele tumour heterogeneity scores

In the cohort, we identified the mutant-allele tumour heterogeneity (MATH) score. The median score was 20.8 (range 8–36) (Additional file 3: Figure S2).

### Effect of smoking

Information on smoking was available for 70 patients with a median number of pack years of 15 (95% CL: 1.5–20 pack years). Thirty patients had never smoked or had less than 10 pack years. The genetic aberrations differed significantly between smokers and non-smokers. For smokers the genes *FGFR2*, *EPHA2* were significantly more likely to be mutated. For non-smokers the genes *IREB*, *BAZ2* and *BRCA1* were significantly more often mutated. The mutational signature for those with low tobacco use was signature 5 and signature 12 in that order. The mutational signature for those with high tobacco use was in contrast 12 and 5. We did not observe any significant difference in MATH score between those with high and low tobacco use. There was also no significant increase in mean number of mutations per sample between these two groups.

## Discussion

Here we present the targeted mutation spectrum of a large cohort of HPV-positive OPSCCs. We corroborate previously reported aberrations (e.g. *FGFR*, *PIK3*, *FAT1*, *NOTCH2*), but also more rare alterations in HPV-positive OPSCCs (e.g. *KALRN*, *KMT2A*, and *SPTBN1*) [11, 20, 32]. Our finding of 3459 mutations with an average of 10 mutations per megabase is higher than that in the TCGA HNSCC cohort [33], which may be explained by the fact that the TCGA cohort is predominately HPV-negative tumours.

Among human cancers, *TP53* is the most frequently mutated gene, and TCGA data has shown that p53 ‘gain-of-functions’ mutants bind to, and upregulate, several chromatin regulatory genes including the methyltransferases MLL1 – also known as *KMT2A* – which is highly prevalent in our cohort (31%) [34]. A chromatin mechanism causal for the progression of tumours with gain-of-function p53 prospects possibilities in the design of combinatorial chromatin-based therapies [34]. Aberrations in *SPTBN1*, that encodes a scaffolding cellular protein involved in the formation of the cytoskeleton, is a useful prognostic biomarker in HPV-negative HNSCCs since patients with tumours expressing *SPTBN1* have four times higher mortality, compared with patients not harbouring this mutation [35]. Although Zhu et al. examines gain-of-function, our data indicates that this gene is also prevalent in HPV+ OPSCCs and could be prognostic in these tumours as well. The known cancer gene *ZNRF3* belonging to the E3 ubiquitin ligase family was also frequent in our cohort (9% of samples). Previously, it has been reported that *ZNRF3* has the ability to inhibit the metastasis and tumorigenesis by suppressing the *Wnt*/β-catenin signalling pathway in nasopharyngeal carcinomas (NPC), hence believed to be a potential molecular target for treatment of NPC. Based on our results, it should also be considered in HPV+ OPSCCs [36]. Due to availability of existing therapeutics and high prevalence of mutations in HPV+ OPSCC, patients with *PIK3CA* and CDK4/CDK6 mutations should be recommend for future phase 0 and I trials. Notably, in our cohort, we merely identified an occurrence of 7 and 1% related to *PIK3CA* pathways and *CCND1* aberrations. Further, the *FGFR2/3* mutations are of particular interest because they were present in 20% of the tumours, in particular the S249C mutation, which is an oncogenic driven in bladder cancer [20]. Upon binding of ligand, *FGFRs* activate a cascade of downstream signalling pathways, such as the mitogen activated protein kinase (*MAPK*), phosphoinositide-3-kinase (PI3K)/Akt pathways signal transducer and activator of transcription (STAT). The mutated *FGFR* isoforms result in oncogenic *FGFR* signalling, promoting tumorigenesis, and the defect *FGFR* signalling pathway is a major contributing factor in the pathogenesis and progression of HNSCC [37, 38]. Inhibition of FGFRs is a promising therapeutic strategy, and phase I and II trials are progressing [39, 40] The tumor suppressor *TRAF3* has previously been brought forward as a potential target for therapy development as it was inactivated in 20% of the HPV-positive tumors in the TCGA cohort. [10] Interestingly, we only identified 5% tumors with this alteration in our cohort.

A previous study [41] has focused on mutations in HPV-positive and HPV-negative patients in a mixed population of larynx, oral cavity, oropharynx and hypopharynx cancer patients. In this study, a high prevalence of *PIK3CA* alterations (mutations and amplifications) was evident, which is not the case in the present study. We speculate that this could be related to 1) a high prevalence of smoking in the present study, 2) ethnical dissimilarities, and 3) the definition of being HPV-positive (RNA-sequencing versus HPV-DNA PCR). These factors could influence the carcinogenic process (smoking vs. no smoking) as well as perhaps not examining the exact same phenotype (being HPV-DNA PCR positive vs. RNA-seq positive for E6/E7), and perhaps tissue specific mutational processes (oropharynx vs. other head and neck cancer subsites).

Other studies evaluating HPV+ OPSCC consistently report mutations in PIK3CA as well as *PTEN, TRAF3*, *NOTCH1.* Although, we did identify these aberrations, they were not among the twenty most prevalent mutations in our cohort. This could be explained by the deduction that these aberrations are not as prevalent in Denmark as other countries, or the fact that our specimens or probes were not adequate set for identifying these mutations.

Interestingly, *IL3RA*, *HLA-A* and *HLA-B* have copy number gains in HPV positive oropharyngeal cancers [3, 42]. Absence of HLA class I (HLA-A and –B) in HPV+ OPSCC has indicated improved outcome which could be used clinically to select patients for trials with de-escalating therapy. The understanding between the genetic background of OPSCC patients and HLA-traits remains incomplete as several HLA-traits have been associated to altered outcome [43, 44].

The landscape of HPV+ and HPV- OPSCCs point to different signatures and structural alterations [11]. To stratify HPV+ from HPV- patients, the most obvious aberrations from our cohort would include *KALRN*, *FGFR2* and *NOTCH2* which are rare in HPV- HNSCC. The *NFE2L2* pathway as well as the promising *PIK3CA* mutation and *CCND1* amplication should also be prioritized.

From the TCGA cohort, the majority of driver mutations were found to be clonal (e.g. “early” mutations opposed to subclonal viewed as “late” mutations) where *CDKN2A* and *TP53* were nearly completely clonal [45]. McGranahan et al. identified three “early” signatures in HNSCC: 1) C > T transitions at CpG sites associated with spontaneous deamination of methylated cytosines; 2) an APOBEC-signature (also seen in “late” mutations); and 3) a smoking-associated signature. In our material, we also identify the APOBEC signature in 23% of the tumors, likewise reported in other HPV+ HNSCCs series [46].

We observed a significant increase in the COSMIC signature 12 associated with certain virus-driven liver cancers. Interestingly, a trial related to these aberrations is progressing. Ribavirin, that target the eIF4E translation initiation factor, is used to treat hepatitis C, and is under investigation for recurrent or metastatic OPSCC (ClinicalTrials.gov, NCT02308241). Ribavirin is also being evaluated along with induction chemotherapy including afatinib (a tyrosine kinase inhibitor) and weekly carboplatin/paclitaxel for stage IV HPV-associated OPSCC (ClinicalTrials.gov, NCT01721525). Additionally for HPV+ OPSCC patients with recurrent or metastatic disease, rigosertib (a *PI3K* (phosphatidylinositol-3 kinase) and *PLK* (Polo-like kinase) signalling pathway inhibitor) is being investigated in a phase II trial (ClinicalTrials.gov, NCT01807546), and in a phase I trial used as initial treatment before platinum based RT-C (ClinicalTrials.gov, NCT02107235). For high risk HPV+ OPSCC patients, the *PI3K* inhibitor, BYL719, is being tested with induction paclitaxel and cisplatin followed by T-site surgery, neck-disssection, and with post-operative risk adapted IMRT (ClinicalTrials.gov identifier, NCT02298595).

Although this study is strengthened by a setup, that includes a deep coverage, a high number of genes, and HPV and p16 status of all patients to ensure HPV-active infections, some limitations should be noted. First, there may be a selection bias both in patients but also because we employed a gene panel. Moreover, the particular selection of genes might influence the findings, since we have included quite large genes (e.g. APOB) where an alteration is more likely opposed to smaller genes. The sub analysis including tobacco merely includes 70 patients due to missing data and a higher number of patients might lead to other findings. Finally, as described in “Methods” section, we strained to reduce artefacts from the use of FFPE tissue (e.g. in the data analysis and tissue preparation) although it should be included as a probable source of bias. Tumor mutational burden (TMB) in the present study was defined as the median number of mutations per megabase examined in the targeted sequencing. As the present study concerns genes previously known to be implicated in carcinogenesis, the TMB would be expected to be higher than studies concerning TMB from e.g. exome-sequencing studies, as there would be fewer mutations per examined base.

When breaking down cosmic signatures from the non-smokers and smokers, we did not observe any difference in enriched cosmic mutational signatures. When looking at mutational signatures in the APOBEC enriched and non-enriched groups, the signature 5 was not enriched in the APOBEC-enriched group, but rather the signature 2. Signature 2 has been attributed to activity of the AID/APOBEC family of cytidine deaminases, and has been related to viral infections.

Both tobacco smoking and defects in DNA repair are known to induce a large number of genetic aberrations, and may be distinct ways to accumulate genetic aberrations required for the emergence of cancer. We found a significant difference between smokers and non-smokers underlining the importance of including tobacco-smoking consumption in prediction models and risk-stratifications. It is likely that the HPV-positive smokers acquire tobacco-related mutations but maintain virus related signatures. Even when stratified on smoking-consumption (none-smokers vs. heavy smokers), a large inter-tumor heterogeneity exists. Thus, if the future aim is to offer a personalized treatment approach it may require a very large battery of anticancer targeted drugs. Although fast-moving technologies have prompted the capability of identifying genetic aberrations promptly and precisely, it remains largely unknown which therapy(−ies) to offer based on the combinations of driver mutations. In order to fuel the development of targeted clinical trials and diagnostic testing, confirmatory studies addressing genetic aberrations in HPV+ OPSCC are needed.

## Conclusion

In conclusion, HPV+ OPSCCs harbour multiple genetic aberrations with both therapeutic and prognostic potential. The discovery of signatures and shared mutations from across organs (i.e. liver, lung and esophagus SCCs) might speed the progress of phase II and III trials, and should be incorporated in drug testing, especially for the heavy smokers.

## Additional files

Additional file 1: **Table S1.** 395 targeted genes. Table of targeted genes. (DOCX 26 kb)

Additional file 2: **Figure S1.** APOBEC mutations. No sample was demonstrated to be significantly enriched for APOBEC mutations, and only a small fraction of mutations in each sample was of the APOBEC type. (JPG 198 kb)

Additional file 3: **Figure S2.** MATH scores. Example of the mutant-allele tumor heterogeneity (MATH) scores, as a measure of tumor heterogeneity. The higher the math-score the higher the tumor heterogeneity. Mid right: Histogram of the MATH-score in the entire cohort. (JPG 201 kb)

## Abbreviations

APOBEC: Apolipoprotein B mRNA editing enzyme, catalytic polypeptide-like
DAHANCA: Danish Head and Neck Cancer group
DPDR: Danish Pathology Data Registry
FDR: False discovery rate
FFPE: Formalin-fixed paraffin embedded
H&E: Hematoxylin and eosin
HNSCC: Head and neck squamous cell carcinoma
HPV: Human papilloma virus
MAPK: Mitogen activated protein kinase
MATH: Mutant-allele tumor heterogeneity
NPC: Nasopharyngeal carcinomas
OPSCC: Oropharyngeal squamous cell carcinomas
PI3K: Phosphoinositide-3-kinase
STAT: Signal transducer and activator of transcription
tCw: Tri-nucleotide DNA sequence
TMB: Tumor mutational burden
