# Supplementary material for: Deep sequencing of human papillomavirus positive loco-regionally advanced oropharyngeal squamous cell carcinomas reveals novel mutational signature
Source: BMC Cancer. 2018 Jun 7;18:640. doi: 10.1186/s12885-018-4567-3 (PMC5992702; doi:10.1186/s12885-018-4567-3)
Supplement: Supplementary file 1 — Table S1. 395 targeted genes. Table of targeted genes. (DOCX 26 kb) [file 12885_2018_4567_MOESM1_ESM.docx]

| 1 | EPHA2 |
| --- | --- |
| 2 | LPHN2 |
| 3 | NRAS |
| 4 | NOTCH2 |
| 5 | BCL9 |
| 6 | MCL1 |
| 7 | SPTA1 |
| 8 | MDM4 |
| 9 | PTPN14 |
| 10 | AKT3 |
| 11 | APOB |
| 12 | DNMT3A |
| 13 | ALK |
| 14 | BIRC6 |
| 15 | MSH6 |
| 16 | SPTBN1 |
| 17 | THSD7B |
| 18 | BAZ2B |
| 19 | ITGB6 |
| 20 | SCN1A |
| 21 | CASP8 |
| 22 | BMPR2 |
| 23 | ERBB4 |
| 24 | FN1 |
| 25 | PER2 |
| 26 | MLH1 |
| 27 | MYD88 |
| 28 | PBRM1 |
| 29 | FOXP1 |
| 30 | EPHA3 |
| 31 | KALRN |
| 32 | PIK3CB |
| 33 | ATR |
| 34 | EPHB3 |
| 35 | FGFR3 |
| 36 | WHSC1 |
| 37 | PDGFRA |
| 38 | KIT |
| 39 | TET2 |
| 40 | FBXW7 |
| 41 | FAT1 |
| 42 | TRIO |
| 43 | MAP3K1 |
| 44 | PIK3R1 |
| 45 | HMGCR |
| 46 | RASA1 |
| 47 | MEF2C |
| 48 | CHD1 |
| 49 | APC |
| 50 | PCDHA11 |
| 51 | PCDHA13 |
| 52 | FAT2 |
| 53 | NPM1 |
| 54 | NSD1 |
| 55 | DSP |
| 56 | CDKN1A |
| 57 | LAMA2 |
| 58 | ENPP1 |
| 59 | FNDC1 |
| 60 | LPA |
| 61 | PLG |
| 62 | EGFR |
| 63 | CDK6 |
| 64 | GNB2 |
| 65 | CUL1 |
| 66 | RHEB |
| 67 | UNC5D |
| 68 | ADAM2 |
| 69 | ASPH |
| 70 | CSMD3 |
| 71 | PARP10 |
| 72 | JAK2 |
| 73 | LINGO2 |
| 74 | PAX5 |
| 75 | TJP2 |
| 76 | GNAQ |
| 77 | PTCH1 |
| 78 | TGFBR1 |
| 79 | SH2D3C |
| 80 | SPTAN1 |
| 81 | Unknown |
| 82 | ABL1 |
| 83 | NOTCH1 |
| 84 | NET1 |
| 85 | SFMBT2 |
| 86 | RET |
| 87 | ARID5B |
| 88 | LGI1 |
| 89 | FGFR2 |
| 90 | MGMT |
| 91 | MUC5B |
| 92 | IPO7 |
| 93 | MICAL2 |
| 94 | WT1 |
| 95 | CKAP5 |
| 96 | MEN1 |
| 97 | USP35 |
| 98 | ATM |
| 99 | KMT2A |
| 100 | IQSEC3 |
| 101 | CCND2 |
| 102 | CDKN1B |
| 103 | ARID2 |
| 104 | KMT2D |
| 105 | MAP3K12 |
| 106 | ERBB3 |
| 107 | CDK4 |
| 108 | MDM2 |
| 109 | IKBIP |
| 110 | APAF1 |
| 111 | CIT |
| 112 | BRCA2 |
| 113 | ZC3H13 |
| 114 | RB1 |
| 115 | FNDC3A |
| 116 | NALCN |
| 117 | NRXN3 |
| 118 | DICER1 |
| 119 | TRAF3 |
| 120 | AKT1 |
| 121 | HERC2 |
| 122 | RASGRP1 |
| 123 | JMJD7-PLA2G4B |
| 124 | SMAD3 |
| 125 | MAN2C1 |
| 126 | SIN3A |
| 127 | IQGAP1 |
| 128 | IGF1R |
| 129 | TSC2 |
| 130 | SLX4 |
| 131 | CREBBP |
| 132 | SMG1 |
| 133 | TAOK2 |
| 134 | MAPK3 |
| 135 | ITGAL |
| 136 | CYLD |
| 137 | MBTPS1 |
| 138 | FAM57A |
| 139 | GEMIN4 |
| 140 | PRPF8 |
| 141 | PLD2 |
| 142 | ZNF594 |
| 143 | TP53 |
| 144 | NCOR1 |
| 145 | FLCN |
| 146 | KIAA0100 |
| 147 | EFCAB5 |
| 148 | NF1 |
| 149 | CDK12 |
| 150 | BRCA1 |
| 151 | CA4 |
| 152 | BPTF |
| 153 | TBCD |
| 154 | SMAD2 |
| 155 | SMAD4 |
| 156 | POLI |
| 157 | NFATC1 |
| 158 | STK11 |
| 159 | SMARCA4 |
| 160 | NOTCH3 |
| 161 | JAK3 |
| 162 | CCNE1 |
| 163 | ARHGAP35 |
| 164 | PPP2R1A |
| 165 | CSNK2A1 |
| 166 | PLCB4 |
| 167 | RALGAPA2 |
| 168 | ASXL1 |
| 169 | PPP1R16B |
| 170 | ARFGEF2 |
| 171 | MC3R |
| 172 | AURKA |
| 173 | RIPK4 |
| 174 | MAPK1 |
| 175 | C22orf31 |
| 176 | NF2 |
| 177 | MYH9 |
| 178 | EP300 |
| 179 | DDX3X |
| 180 | KDM5C |
| 181 | AR |
| 182 | MED12 |
| 183 | HCFC1 |
| 184 | ERRFI1 |
| 185 | ARID1A |
| 186 | AGO3 |
| 187 | MPL |
| 188 | FUBP1 |
| 189 | OLFML2B |
| 190 | DDR2 |
| 191 | F5 |
| 192 | SELP |
| 193 | KCNT2 |
| 194 | ZC3H11A |
| 195 | MAP4K3 |
| 196 | XPO1 |
| 197 | NCKAP1 |
| 198 | VHL |
| 199 | RAF1 |
| 200 | TGFBR2 |
| 201 | ALS2CL |
| 202 | RHOA |
| 203 | BAP1 |
| 204 | MITF |
| 205 | PARP9 |
| 206 | UROC1 |
| 207 | TBL1XR1 |
| 208 | PIK3CA |
| 209 | SLIT2 |
| 210 | KDR |
| 211 | PCDH18 |
| 212 | TENM2 |
| 213 | KCNK16 |
| 214 | RP11-321N4.5 |
| 215 | ESR1 |
| 216 | ARID1B |
| 217 | MET |
| 218 | EZH2 |
| 219 | MSR1 |
| 220 | FGFR1 |
| 221 | PKHD1L1 |
| 222 | BNC2 |
| 223 | SYK |
| 224 | TSC1 |
| 225 | CELF2 |
| 226 | CCAR1 |
| 227 | ABTB2 |
| 228 | DAGLA |
| 229 | NOX4 |
| 230 | C1S |
| 231 | TM7SF3 |
| 232 | GALNT6 |
| 233 | TSPAN31 |
| 234 | ANKS1B |
| 235 | FLT3 |
| 236 | ELF1 |
| 237 | TFDP1 |
| 238 | FBLN5 |
| 239 | IDH2 |
| 240 | CIITA |
| 241 | CHD9 |
| 242 | CTCF |
| 243 | CDH1 |
| 244 | MYO1C |
| 245 | WRAP53 |
| 246 | MAP2K4 |
| 247 | ERBB2 |
| 248 | RARA |
| 249 | RNF43 |
| 250 | GH2 |
| 251 | ZNF750 |
| 252 | MAP2K2 |
| 253 | AKT2 |
| 254 | NLRP12 |
| 255 | GNAS |
| 256 | ABCG1 |
| 257 | CRKL |
| 258 | SMC1A |
| 259 | ALAS2 |
| 260 | ATRX |
| 261 | OCRL |
| 262 | ARHGEF6 |
| 263 | HNRNPCL1 |
| 264 | IRF6 |
| 265 | MSH2 |
| 266 | BCL11A |
| 267 | CTNNB1 |
| 268 | IFRD2 |
| 269 | PDE12 |
| 270 | TRA2B |
| 271 | PCDHGA8 |
| 272 | PCDHGA12 |
| 273 | SMO |
| 274 | BRAF |
| 275 | CLVS1 |
| 276 | SNAPC3 |
| 277 | PSIP1 |
| 278 | CDKN2A |
| 279 | RP11-145E5.5 |
| 280 | CDKN2B |
| 281 | HRAS |
| 282 | QSER1 |
| 283 | CATSPER1 |
| 284 | TSKU |
| 285 | RAB35 |
| 286 | DLST |
| 287 | BUB1B |
| 288 | B2M |
| 289 | IREB2 |
| 290 | CCDC40 |
| 291 | TRIP10 |
| 292 | CLIP3 |
| 293 | ERCC2 |
| 294 | NCR1 |
| 295 | ZNF835 |
| 296 | COL6A2 |
| 297 | KDM6A |
| 298 | PLXNB3 |
| 299 | SRPK3 |
| 300 | RENBP |
| 301 | AC010127.3 |
| 302 | WIPF1 |
| 303 | CUL3 |
| 304 | MKRN2 |
| 305 | GNL3 |
| 306 | PIM1 |
| 307 | CCND3 |
| 308 | CCND1 |
| 309 | ATN1 |
| 310 | RP1-127H14.3 |
| 311 | CCDC64 |
| 312 | NFKBIA |
| 313 | LCMT2 |
| 314 | ADAL |
| 315 | GAA |
| 316 | CEBPA |
| 317 | SMARCB1 |
| 318 | NCF2 |
| 319 | SLC4A1AP |
| 320 | NFE2L2 |
| 321 | KCNIP4 |
| 322 | KCNK17 |
| 323 | TMEM176B |
| 324 | PEX2 |
| 325 | RSPO2 |
| 326 | MYC |
| 327 | PTEN |
| 328 | CHD4 |
| 329 | C12orf57 |
| 330 | LYRM5 |
| 331 | KRAS |
| 332 | MYZAP |
| 333 | MAP2K1 |
| 334 | SUPT4H1 |
| 335 | FAM210B |
| 336 | ZNRF3 |
| 337 | KPRP |
| 338 | ALS2CR12 |
| 339 | IDH1 |
| 340 | DTX3L |
| 341 | PACRGL |
| 342 | NOP2 |
| 343 | NLRP13 |
| 344 | NLGN3 |
| 345 | ELF4 |
| 346 | NKX2-1 |
| 347 | SMG7 |
| 348 | SYNCRIP |
| 349 | RAC1 |
| 350 | NTHL1 |
| 351 | ZNRF4 |
| 352 | RPSAP58 |
| 353 | ARAF |
| 354 | PCDHA2 |
| 355 | TMEM176A |
| 356 | PABPC3 |
| 357 | POLR2M |
| 358 | CTD-3157E16.1 |
| 359 | GNA11 |
| 360 | NAA10 |
| 361 | WDR34 |
| 362 | ENO2 |
| 363 | C1orf74 |
| 364 | PCDHGB3 |
| 365 | OR9A4 |
| 366 | RP11-45M22.4 |
| 367 | CSDE1 |
| 368 | SDCCAG8 |
| 369 | TAF8 |
| 370 | GAB2 |
| 371 | XRN1 |
| 372 | OR5L1 |
| 373 | CPB2 |
| 374 | UGT2B11 |
| 375 | PGAP3 |
| 376 | PKD1 |
| 377 | N4BP2L1 |
| 378 | RILP |
| 379 | PRKAG1 |
| 380 | PAK6 |
| 381 | SSH2 |
| 382 | LSMEM2 |
| 383 | TOR2A |
| 384 | CCNH |
| 385 | TBC1D16 |
| 386 | RPS6KL1 |
| 387 | PCDHA9 |
| 388 | KCTD21 |
| 389 | FIP1L1 |
| 390 | ALKBH6 |
| 391 | HLA-B |
| 392 | TARBP2 |
| 393 | TC2N |
| 394 | ZBED6 |
| 395 | HLA-A |
