# Supplementary figures and images for: Deep sequencing of human papillomavirus positive loco-regionally advanced oropharyngeal squamous cell carcinomas reveals novel mutational signature
Source: BMC Cancer. 2018 Jun 7;18:640. doi: 10.1186/s12885-018-4567-3 (PMC5992702; doi:10.1186/s12885-018-4567-3)

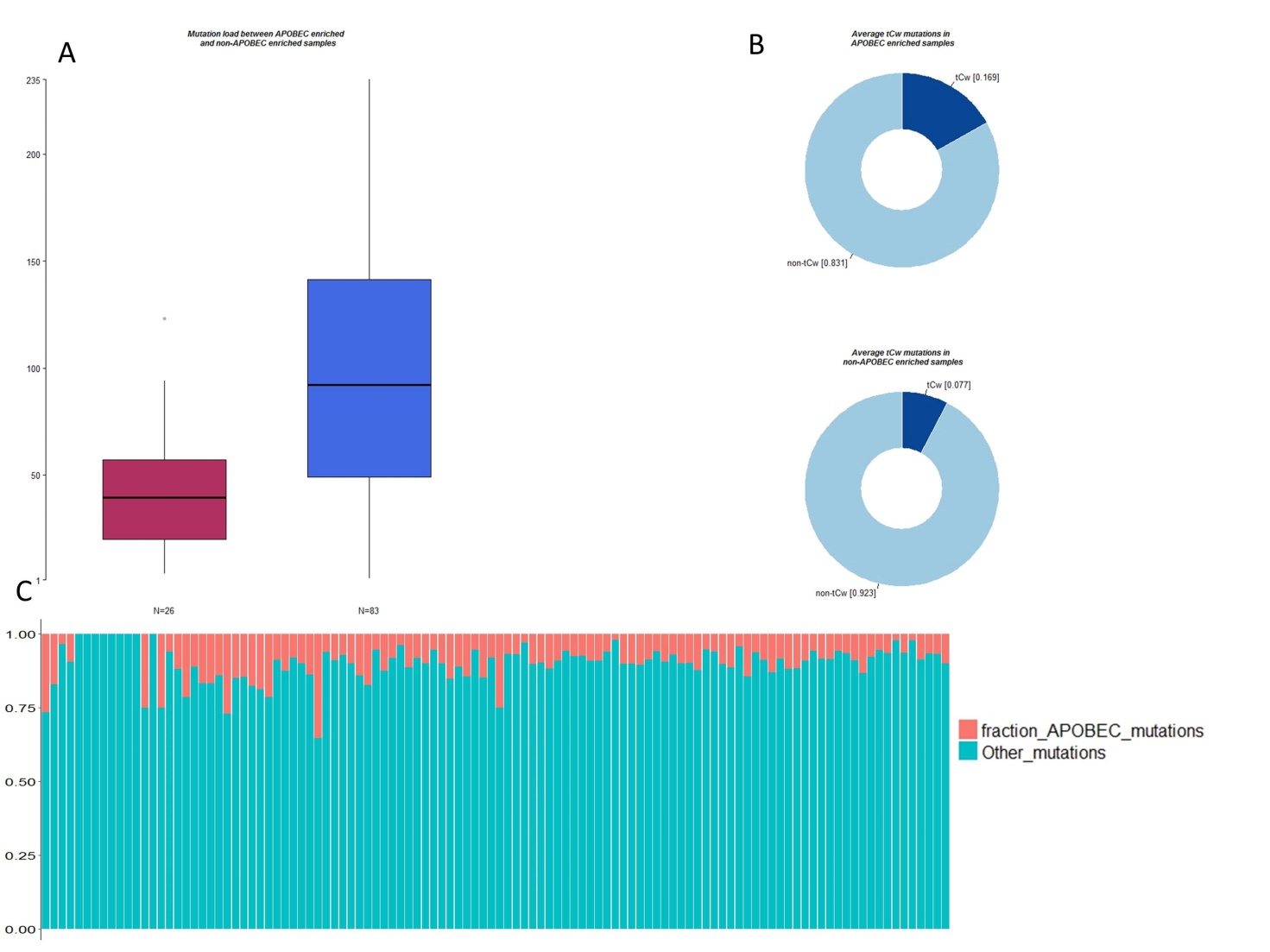

Supplement: Supplementary file 2 — Figure S1. APOBEC mutations. No sample was demonstrated to be significantly enriched for APOBEC mutations, and only a small fraction of mutations in each sample was of the APOBEC type. (JPG 198 kb) [file 12885_2018_4567_MOESM2_ESM.jpg]

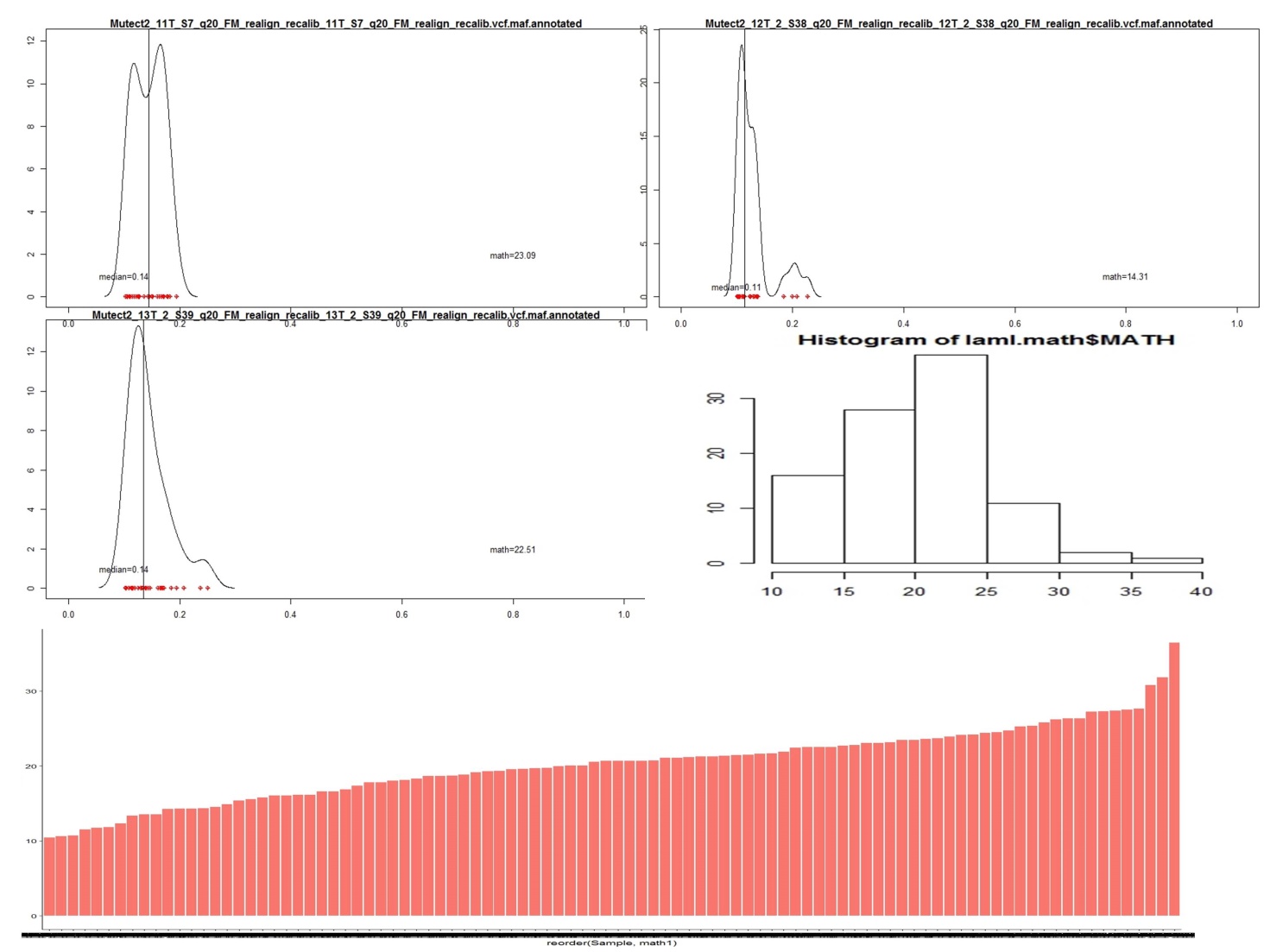

Supplement: Supplementary file 3 — Figure S2. MATH scores. Example of the mutant-allele tumor heterogeneity (MATH) scores, as a measure of tumor heterogeneity. The higher the math-score the higher the tumor heterogeneity. Mid right: Histogram of the MATH-score in the entire cohort. (JPG 201 kb) [file 12885_2018_4567_MOESM3_ESM.jpg]
